# Evaluating the Release of Different Commercial Orally Modified Niacin Formulations In Vitro

**DOI:** 10.3390/polym15143046

**Published:** 2023-07-14

**Authors:** Christiane Chbib, Md. Abdur Rashid, Sarthak M. Shah, Mohsin Kazi, Mohammad N. Uddin

**Affiliations:** 1 Department of Pharmaceutics, College of Pharmacy, Larkin University, 18301 N Miami, Miami, FL 33169, USA; cchbib@larkin.edu; 2 Department of Pharmaceutics, College of Pharmacy, King Khalid University, Al Faraa, Abha 62223, Saudi Arabia; 3 Department of Pharmaceutics, College of Pharmacy, Mercer University, 3001 Mercer University Drive, Atlanta, GA 30341, USA; sarthak.modi.shah@live.mercer.edu; 4 Department of Pharmaceutics, College of Pharmacy, King Saud University, P.O. Box 2457, Riyadh 11451, Saudi Arabia; mkazi@ksu.edu.sa

**Keywords:** niacin (nicotinic acid), modified-release tablets, in vitro dissolution studies, quantification, bioavailability, UV spectroscopy

## Abstract

Objectives: To evaluate the release profile of different modified-release oral formulations of niacin, such as immediate-release (IR) powder and tablets, timed-release (TR) caplets, extended-release (ER) capsules, and controlled-release (CR) tablets, to assure their defined release pattern and correlate this release with their matrix polymers. Significance: Niacin is used to manage hyperlipidemia by reducing cutaneous flushing and hepatotoxicity adverse events. The release profiles of different types of modified-release dosage forms depend on the types of coating materials (polymers) used in the matrix formation. Although different types of niacin formulations exist, none of the niacin dissolution profiles have been evaluated and compared in the literature. Methods: Four commercial orally modified-release niacin brands were collected from a local CVS pharmacy retail store, in Miami, FL, USA. The in vitro release study was conducted in simulated gastric fluid (SGF) and simulated intestinal fluid (SIF) conditions. Results: The results of the release patterns of four niacin-modified dosage forms (IR, ER, TR, and CR) were aligned with their release definitions. However, the CR dosage form did not follow an ideal release pattern. Conclusions: The release rate of niacin in vitro was pH dependent, which was confirmed by the similarity factor (f2) results. All the f2 comparison values were below 50 in both the SIF and SGF media, while all the comparisons were below the f2 values for all brands in the SIF media.

## 1. Introduction

**Figure 1:**
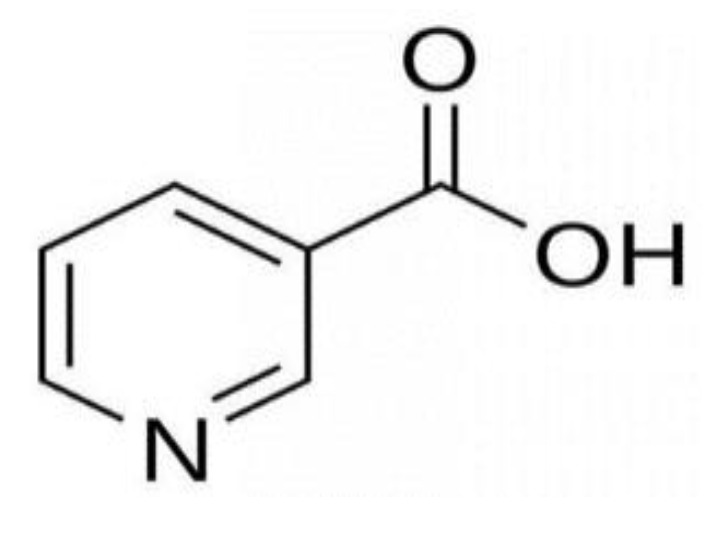
Chemical structure of vitamin B3 or niacin.

Niacin (vitamin B3), shown in Figure 1, is known as nicotinic acid and is taken as a dietary supplement by adults [1]. In the gastrointestinal tract, niacin is quickly and easily absorbed from the intestinal tract and hepatically metabolized in the liver before eventually being distributed extensively to the tissues in the body. Here, it is converted to its active coenzyme forms, nicotinamide adenine dinucleotide, and nicotinamide adenine dinucleotide phosphate. Its absorption varies depending on the dosage forms: for oral dosing, an immediate-release form will reach peak serum concentration within 45 min of ingestion, while an extended-release form will peak 4–5 h after ingestion [2]. Pharmacologically, niacin is considered an anti-dyslipidemia agent and is used to manage hyperlipidemia when administered in combination with other anti-dyslipidemia agents, as recommended by the National Cholesterol Education Program Adult Treatment Panel III (NCEP) [3,4]. Niacin has been shown to increase high-density lipoprotein cholesterol (HDL-C) levels by inhibiting the hepatic uptake of apolipoprotein A-1 and reducing HDL clearance [5]. The National Institute of Health (NIH) has identified several groups at risk of niacin insufficiency among individuals who are undernourished or have Hartnup disease, or carcinoid syndrome. In addition, individuals who do not consume the vital nutrients riboflavin, pyridoxine, and iron are also at risk of niacin deficiency [6]. Niacin was shown to elicit several positive effects on ulcerative colitis (UC) in mice and humans by activating the prostaglandin (PG) D2/D prostanoid receptor 1 axis. Specifically, vascular permeability improved, the number of epithelial cells undergoing apoptosis decreased, and niacin treatment aided mucosal healing in patients with moderate UC [7]. It is available over the counter in major pharmacies (chemist shops) in the United States, mostly in capsule and tablet forms. It does not need a prescription to be dispensed and is categorized as a dietary supplement that follows the Dietary Supplement Health and Education Act of 1994, allowing its sale without FDA approval. Additionally, the dietary supplement category needs to meet only the minimum good manufacturing practices (GMP) to be allowed on the market.

Since these products are marketed as supplements, they are sold without any conducted pre-formulation dissolution studies [8]. Dissolution studies are an important test for all solid oral dosage forms. A suboptimal therapeutic window could potentially lead to low bioavailability leading to reduced efficacy. Conversely, a high bioavailability can produce unwanted adverse toxic effects, such as hepatotoxicity and skin flushing [9]. Dissolution studies of ER formulations have shown a large variability among available sustained-release formulations [10,11]. Niacin is currently available in the market as IR and ER formulations. Variations in the modified release formulation impact niacin’s dissolution rate, thereby affecting safety profiles. The brands of niacin selected in this study reflect the different oral modified release formulations of niacin, such as IR powder, IR tablet, timed-release caplet, ER capsule, and CR tablet.

Modified-release niacin is different from IR in terms of the delay in the drug delivery after administration at less frequent intervals. Long-acting (LA) dosages can be sustained, controlled release, or timed release. Sustained release (SR) dosage forms are designed to release the drug at a predetermined rate to maintain a constant drug concentration. This dosage form ensures minimum side effects and maximum efficiency [12]. CR maintains drug release over a sustained period at a nearly constant rate. CR significantly reduces the dose frequency. TR niacin is accomplished over a period of 8 h. ER is an intermediate release of IR and LA. The different patterns in the release profiles of the modified niacin dosage forms depend on their excipient compositions. Mainly, different types of polymers used in formulations have different dissolution rates in solution based on the pH value, such as in the stomach or large and small intestines. This study assesses the impact of pH on the niacin dissolution track.

**Scheme 1:**
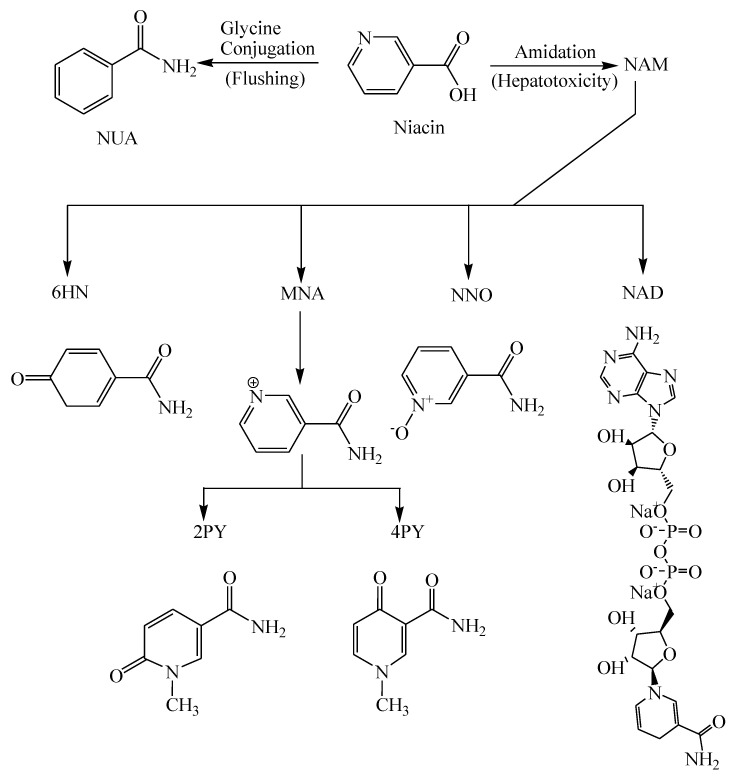
Primary (amidation) and secondary (conjugation) pathways for niacin metabolism. NUA: nicotinuric acid; niacin: nicotinic acid; NAM: nicotinamide; 6 HN: 6-hydroxynicotinamide; MNA: N-methylnicotinamide; NNO: nicotinamide-N-oxide; NAD: nicotinamide adenine dinucleotide; 2 PY and 4 PY: metabolites of pyridone.

**Figure 2:**
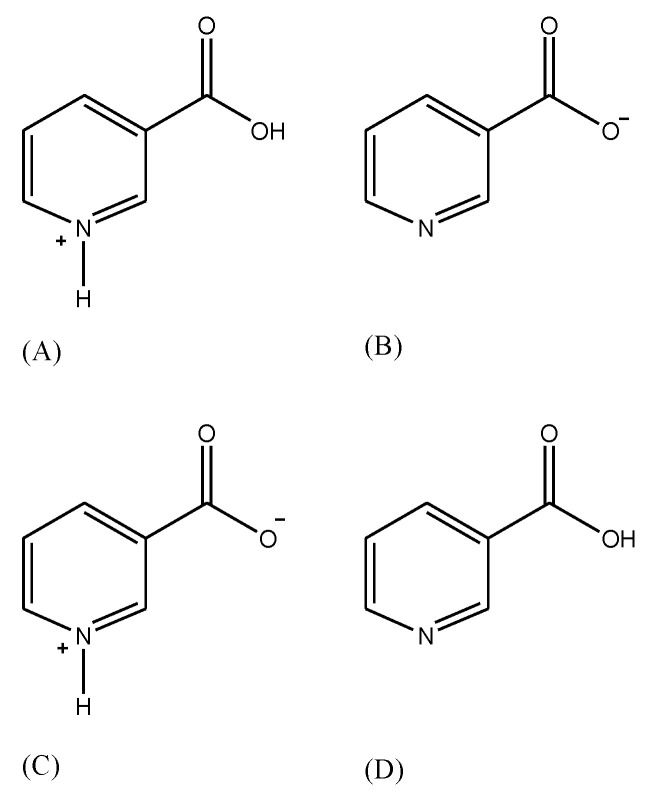
Scheme shows the four possible forms of niacin (or nicotinic acid); (**A**) conjugate acid predominates in acidic solution, (**B**) conjugate base occurs as minor form in aqueous media, (**C**) zwitterion predominates in aqueous media, (**D**) uncharged predominates in basic solution.

The rationale for developing modified release forms of niacin lies in its metabolism process (Scheme 1) in the body. Niacin undergoes extensive first-pass metabolism in the liver [13]. The conjugative pathway results in the formation of metabolites, such as nicotinuric acid (NUA), a glycine conjugate of niacin that is responsible for the vasodilation and flushing adverse effects of niacin [4]. The amidation pathway, described as high affinity and low capacity, is associated with the hepatotoxicity side effect of niacin. The rapid dissolution of the IR niacin saturates the amidation pathway, leading to the saturation of the conjugative pathway and resulting in higher rates of flushing and hepatotoxicity adverse events, which is the reason for the modified slower release forms of niacin being developed. SR niacin is metabolized via the conjugative pathway [14]. The predominant metabolite formed after the administration of IR niacin products is nicotinuric acid, while after SR formulations, various products of nicotinamide biotransformation exist [15]. In addition to the metabolic pathways, the release of the niacin drug from the delivery system depends on its chemical structure, which impacts solubility; hence, the dissolution process. Niacin is an ionized active compound. It can exist in four possible forms: conjugate acid, which predominates in acidic solution; conjugate base, which occurs as a minor form in aqueous media; zwitterion, which predominates in aqueous media; uncharged molecule, which predominates in basic solution (Figure 2). The solubility of niacin is pH dependent, with slower dissolution in water and faster dissolution in acidic media, such as 0.1 N HCl [16]. Therefore, the solubility of the drug will be different in the stomach at pH 1.2 and intestine at pH 6.8. All the modified dosage forms of niacin are administered orally and it is absorbed primarily in the small intestine, with some also absorbed in the stomach [14]. Thus, the niacin drug encounters two different acidic environments, one in the stomach with a pH of 1.2, and afterward, in the intestines at pH 6.8. Therefore, it will be very informative to assess the dissolution of different dosage forms in the two different pH environments of the stomach and intestine. The objective of this study was to (1) evaluate the dissolution profiles of the different niacin dosage formulations being marketed in the USA; (2) track the correlation between the different dosage forms and the two major clinical limitations of niacin cutaneous flushing mainly associated with the IR formulation and hepatotoxicity mainly associated with the SR formulation [17].

Niacin dissolution is pH-dependent: Niacin is an ionized active compound. Its solubility is pH-dependent with slower dissolution in water and faster in acidic media, such as in 0.1 N HCl [17].

## 2. Materials and Methods

### 2.1. Materials

Four commercial orally modified release niacin brands, IR (250 mg), ER (500 mg), TR (1000 mg), and SLO (500 mg), were purchased from a local CVS pharmacy retail store in Miami, FL, USA. All batches for every drug were selected to have a close expiration date. The different types of modified niacin dosage forms that were used as investigated medicinal products (IMPs) in this study are listed in Table 1, with their formulation, drug amount, lot number, expiration date, manufacturer, and excipients. Niacin crystalline, white powder (high HPLC purity 99.97%) was purchased from bulksupplements.com. All the reagents used were analytical grade. The reagents ACS (American Chemical Society)-grade SGF without pepsin (Fisher Scientific catalog #7108-16; pH 1.2), SIF without pancreatin (Fisher Scientific catalog #7109-16; pH 6.8), ACS-grade distilled water, and ACS-grade NaCl slats were obtained from VWR chemical suppliers, Fl, USA. To complete the experiments, the following was used (1) the dissolution system: Distek 2100A, with TCS 0200C Temperature Control System, 2230A Dissolution Sampler, and 2230A Sample Pump. (2) UV spectrophotometer: VWR 3100PC, Model: ULC1610018, with matched 1 cm quartz cells. (3) Fisher brand™ pH Pen as a pH meter.

### 2.2. Methods

#### 2.2.1. In Vitro Dissolution Study

A dissolution study was performed in vitro using a Distek Dissolution System (Model 21000 C) type I (USP Dissolution apparatus, Distek, North Brunswick, NJ, USA) with 40 mesh rotating mini baskets. Dissolution testing was carried out to measure the rate of drug release from the given dosage forms. Throughout the study, a temperature of 37 ± 0.5 °C was maintained using a TCS 0200 C Temperature Control System, while USP Apparatus 2 (paddles) was used for constant stirring at 90 rpm.

#### 2.2.2. Experimental Methods

The dissolution vessels were filled with 900 mL of the SGF and SIF solvents to reach a maximum concentration of 0.5556 g/L for regular niacin, SLO niacin, and ER niacin and 1.111 g/L for TR capsules. Using a 2230 A Dissolution Sampler, samples were analyzed at 5, 15, and 30 min and 1, 2, 4, 6, 8, 10, 12, 18, and 24 h. All four dosage forms were first tested in stimulated gastric fluid and after in stimulated intestinal fluid to mimic the gastrointestinal tract system.

#### 2.2.3. UV Spectrophotometer Analysis

After dilution, 3 mL of the samples from the SGF and SIF were placed in a UV/VIS spectrophotometer using quartz cuvettes to obtain maximum absorbances of 219 nm and 262 nm. After obtaining reference curves, a dissolution study was performed for each brand. Experiments were run in triplicates. The absorbance and wavelength were recorded. The concentration was calculated for each niacin formulation.

#### 2.2.4. Comparison between Dissolution Profiles Using the f2

A comparison between different dissolution profiles for each oral modified release commercial tablet was conducted using f2, which was determined according to Equation (1) [18]. The major advantages of the f2 equation are that it is easy to compute, and it provides a single number to describe the comparison of dissolution profile data.

(1) $$f2=50\;Log\;\left\{{\left[1+\frac{1}{n}\sum_{t=1}^{n}W_{t}{\left(R_{t}-T_{t}\right)}^{2}\right]}^{-0.5}\times 100\right\}$$

where

*n* is the number of sample points

*W_t_* is the optional weight factor

*R_t_* is the reference assay at time point *t*

*T_t_* is the test assay at time point *t*

## 3. Results

### 3.1. Determining Niacin Calibration Curves for Concentration Calculation

Niacin solutions were prepared by dissolving each niacin brand in the previously mentioned reaction media, at a concentration range of 5 × 10^−7^ to 1 × 10^−2^ g/L. The reference solutions were scanned using a wavelength region of 190 to 400 nm. The absorbance was observed against the niacin-free solutions of the reaction media (blank). Two absorption peaks were found for niacin in each reaction medium, at 219 and 262 nm. The obtained data were used to draw the calibration curve for both wavelengths.

(2) $$A=\frac{V_{max}\times c}{K_{m}+c}$$

where *A* is the absorption, *c* is the concentration, and *K_m_* and *V_max_* are fitting parameters. All fittings were iterated using OriginPro 2018 (OriginLab Corporation, “OriginPro, Version 2018”. Northampton, MA, USA) and applied to the orthogonal distance regression algorithm [7].

**Figure 3:**
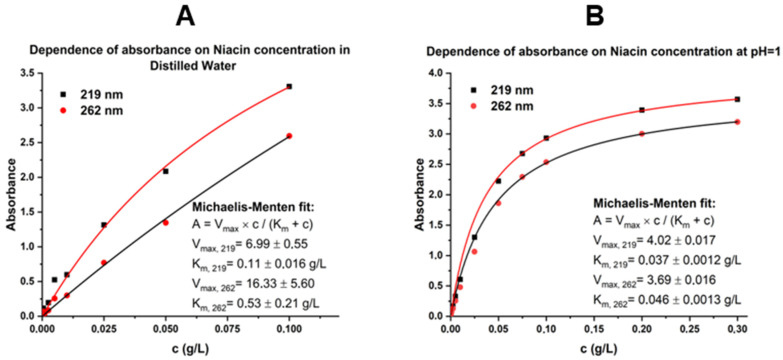
Michaelis–Menten fits and its parameters for the dependence of absorbance on niacin concentration at pH 1 and pH 7.

By using the Michaelis–Menten (M–M) equation, R2 > 0.99 was obtained using two fitting parameters for both wavelengths in both reaction environments. The measurements were carried out until both wavelengths approached Abs = 3, corresponding to 0.1 g/L in SIF and 0.3 g/L at SGF. The linear equation in Panel A of Figure 3 with λ = 262 nm, describes the correlation between absorbance and concentration at a high pH and wavelength. While a correlation can be found between absorbance and concentration at both wavelengths, it is visible that at λ = 219 nm, absorbance outsteps 3 at a significantly lower concentration compared to when λ = 262 nm, which results in less reliable data. Thus, for evaluation purposes, only data belonging to the larger wavelength will be discussed. The obtained equations and fitting parameters were used to obtain unknown concentrations of niacin throughout the dissolution study.

### 3.2. Drug Concentration Measurement

To obtain the niacin concentration at each measurement point, the dilution factor, and the volume loss due to sampling were considered. Concentration-based absorbance was calculated from the absorbance of the sample by using the fitting parameters of the M–M equations. The concentration was obtained by Equation (3):

(3) $$c=\frac{c_{d}\times (900\;\mathrm{mL}-\left(n-1\right)\times 3\;\mathrm{mL})}{1000\;\mathrm{mL}}$$

where *c_d_* is the concentration after dilution, 900 mL is the total volume used in the experiments, *n* is the number of actual samples, and 3 mL is the volume loss due to auto-sampling. To quantitatively compare the release rates of the different niacin formulas, the concentrations were converted to percentages of the timely concentration of the maximum reachable concentration.

### 3.3. Release Profiles of Different Niacin Tablet Formulations in SGF and SIF

**Figure 4:**
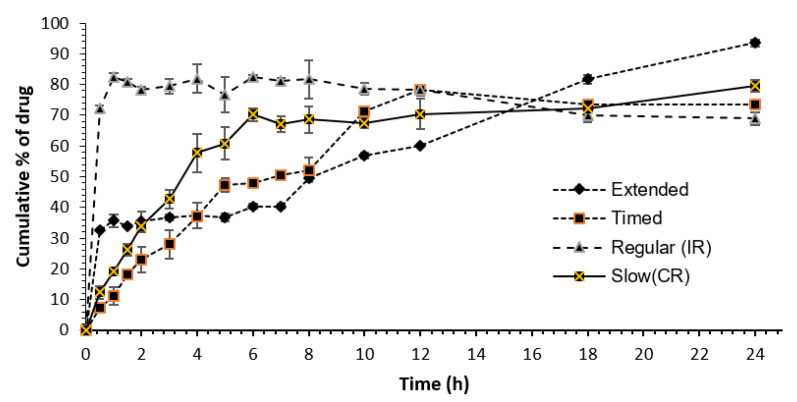
Release profiles of four different formulations: extended, timed, regular, and SLO niacin at pH 1 for 24 h.

Four different niacin drug formulations displayed varying release patterns in SGF and SIF. Regular niacin has an IR dosage form, it is released immediately in this formulation; therefore, the maximum concentration should be reached within a short period of time (immediately). Results show (Figure 4) that the drug concentration was high in the short term in both media. In SGF, 100% of the drug was released after 2 h hh of dissolution. However, the maximum reached concentration was found to be 80% of the niacin concentration.

**Figure 5:**
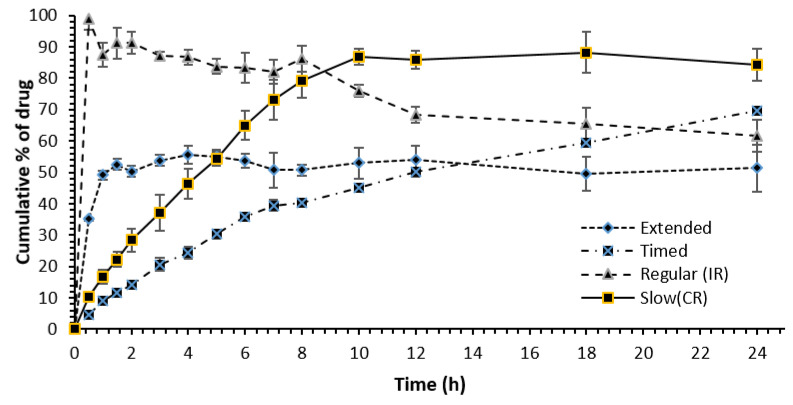
Release profile of four different formulations, including extended, timed, regular, and slow niacin at pH 7 for 24 h.

**Figure 6:**
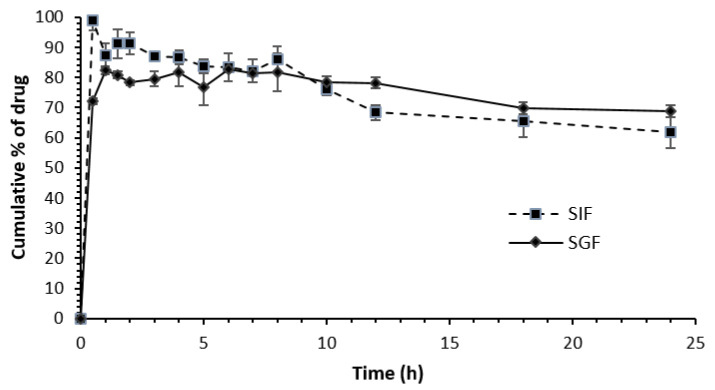
Release profile of regular or immediate release formulation of niacin at pH 1 and pH 7.

**Figure 7:**
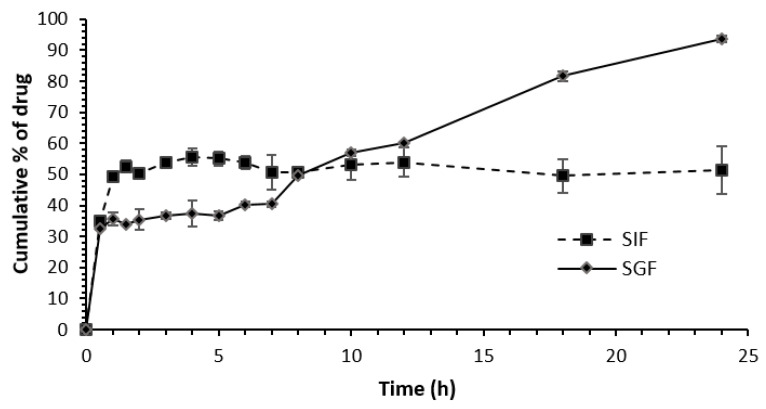
Release profile of extended formulation of niacin in pH 1 and pH 7.

ER niacin showed that most of the drug was released within 2 h in both media. However, in SIF (Figure 5 and Figure 6), this formulation never reached 100% release; instead, it remained constant at approximately 50% niacin concentration release over a 24 h period. On the other hand, the formulation released 100% of the niacin concentration in SGF at 24 h (Figure 6). The slow and constant release of this formulation in SGF indicates that this formulation is designed to slowly release the drug at low pH (similar to the stomach conditions), and higher pH values (similar to the intestinal conditions) (Figure 7).

**Figure 8:**
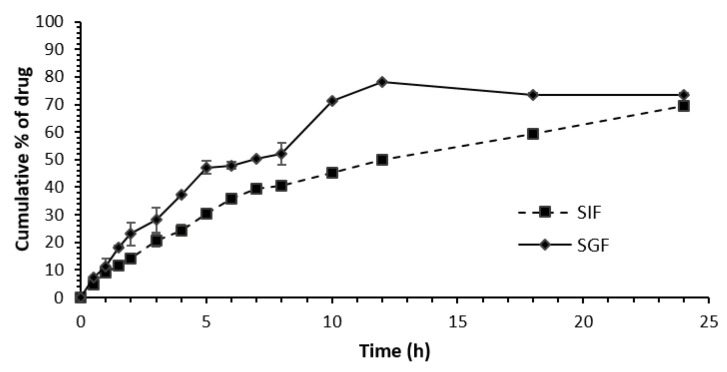
Release profile of timed-release formulation of niacin in pH 1 and pH 7.

TR niacin shows that most of the drug was released in both media within 2 h. However, in SIF, this formulation never reached 100% concentration release; instead, it remained constant for up to 24 h. On the other hand, the formulation released the highest niacin concentration in SGF at 24 h (Figure 8). The slow and constant release rate of this formulation in SGF indicates that it is designed to slowly release the drug at low pH levels consistent with conditions in the stomach area and exerts extended release at higher pH levels, which is consistent with the intestinal conditions. The release pattern in SGF is not constant, but in SIF, it displays a more consistent and homogeneous drug release over time. It is worth noting that at both pH values, the maximal niacin concentration release was approximately 65–70%. By using a proprietary, natural wax-matrix coating technology, TR niacin is released slowly over a 6–8 h period to reduce flushing.

**Figure 9:**
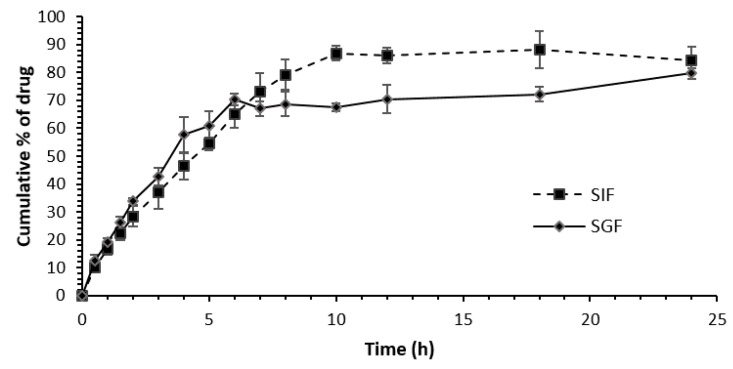
Release profile of SLO formulation of niacin in pH 1 and pH 7.

**Figure 10:**
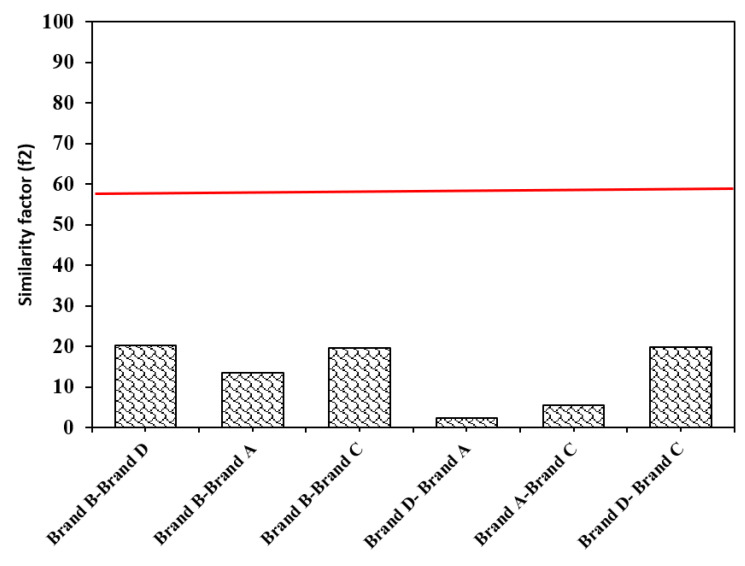
Comparison of niacin dissolution profiles in SGF using f2.

**Figure 11:**
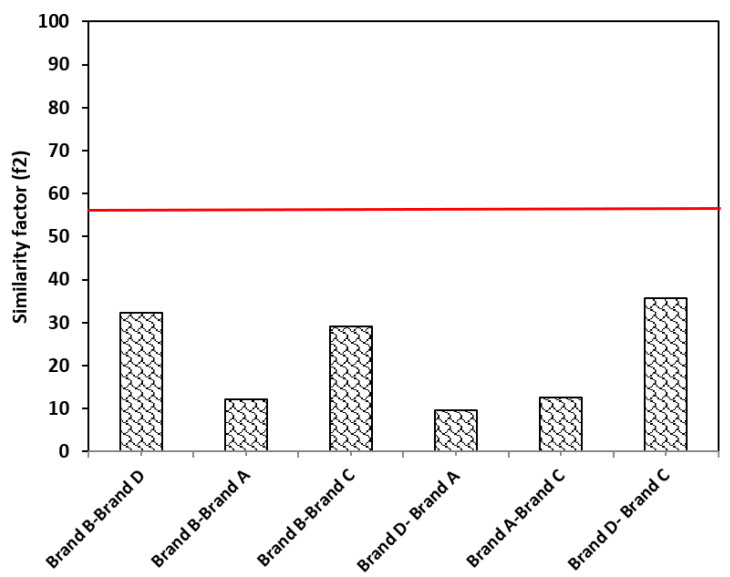
Comparison of niacin dissolution profiles in SIF using f2.

The SLO niacin tablets were prepared using a polygel controlled-release delivery system. This formulation confirms the gradual and measured release of niacin (nicotinic acid) and is designed to reduce the incidence of flushing and itching commonly associated with niacin use. In the dissolution study, SLO niacin or CR in SGF, the drug concentration increased until reaching a maximum at 6 h. Afterward, the concentration remained constant, which indicated that no additional niacin was released. In SIF, the niacin dissolution increased to a maximum concentration of 50% at 5 h before remaining constant, which similarly indicates that niacin is not further released after 6 h of dissolution (Figure 9). The in vitro f2 was calculated for every pair of branded commercial niacin tablets in both SIF and SGF media and is shown in Figure 10 and Figure 11. The similarity factor results for both SIF and SGF were consistent, i.e., they were not bioequivalent (f2 < 50).

## 4. Discussion

Niacin (nicotinic acid), a water-soluble vitamin, is sold as an over-the-counter supplement and also as a prescribed drug. Niacin acts as a precursor to the synthesis of the coenzymes NAD and NADP, which are involved in important reactions that maintain the redox state of cells (e.g., glycolysis and pentose phosphate shunt) [19,20]. In pharmacological doses, niacin reduces plasma triglycerides, cholesterol, and atherogenic apolipoprotein B (apoB)-containing lipoproteins and increases antiatherogenic apoA-I-containing high-density lipoprotein levels, thereby preventing atherosclerotic cardiovascular disease [21,22]. Therefore, the absorption of this drug in the human body is an important factor. However, the mechanism of niacin uptake was not fully understood until Svetlana et. al. conducted a study using human-derived intestinal epithelial Caco-2 cells and purified intestinal brush border membrane vesicles (BBMVs), isolated from human organ donors, to assess niacin [23]. The result showed that niacin uptake by Caco-2 cells is dependent on several factors but mostly on extracellular acidic pH. Currently, there are several forms of niacin that exist on the market as OTC products; however, the study of their dissolution has yet to be conducted. The dissolution study predicts the extent of absorption of the different dosage forms, and hence, the bioavailability and efficacy of the drug. Therefore, it is very important to conduct the dissolution study on the different niacin dosage forms to assess whether the drugs sold as OTC products are effective and the extent to which the drugs are being absorbed by the body.

This study compares the dissolution profiles of different niacin dosage forms found on the US market. Poon et al. [24,25] studied the niacin dissolution rate in 2006 by only employing an acidic media in vitro. The study’s major limitation was that the GI conditions were inconsistent. In this project, the experiments were first performed in acidic media, and then, in neutral media to reflect the in vivo gastric and intestinal conditions. In vitro evaluation found that Brand A, Brand D, and Brand C were aligned with their pharmacokinetic release definitions, while Brand B was the exception. IR usually follows the conjugation pathway for metabolism, which leads to the flushing adverse side effect in patients. This formulation releases niacin very quickly, within 30–60 min. LA formulations are slowly absorbed by the human body. These formulations are metabolized via the nicotinamide pathway, which can cause hepatotoxicity. The intermediate formulation, ER, has been shown to dampen the adverse effects of niacin by reducing flushing and hepatotoxicity. Extended-release formulations are usually absorbed over 8–12 h [25].

Polymer(s) of the tablet matrix or capsules could play a vital role in drug delivery systems. The different types of modified release forms depend on their polymers. These phenomena depend upon the interaction between the dissolution media, polymeric matrix, and drug molecule. Polymers including polyvinyl-pyrrolidone (PVP) and hydroxypropyl methylcellulose (HPMC) are commonly used in immediate-release tablet formulations as binders to aid the formation of granules that improve the flow and compaction properties of tablet formulations prior to tableting [26]. The most commonly used water-insoluble polymers for extended-release applications are ammonium ethacrylate copolymers (Eudragit RS and RL), cellulose derivatives ethylcellulose, cellulose acetate, polyvinyl derivative, and polyvinyl acetate. A summary list of different excipient types of modified release forms of niacin is provided in Table 1, which correlates to the drug release behavior.

The release pattern for each formulation can be explained by considering the ingredients in each brand. Brand A, manufactured by General Nutrition Corporation (GNC), has several components, such as cellulose and dicalcium phosphate. Cellulose, which forms the film coat of the tablet caused an immediate release in this study. Excipient dicalcium phosphate is the filler in the dosage form. Since cellulose and dicalcium phosphate are soluble at a lower pH, this may explain the high release rate. In addition, both components are not water soluble at a high pH [27], which may explain why the niacin release is decreased, consistent with our findings. Brand A showed a higher niacin release rate from 0 to 4 h under SGF and a low release rate after 4 h under SIF media. The fast niacin release has been linked to the saturation of the amidation pathway in humans resulting in flushing and vasodilation [28]. 

The slow or controlled release should exhibit a slow niacin release in the body. This tablet, manufactured by main Pointe Pharmaceuticals, LLC, consisted of several ingredients: hypromellose, silicon dioxide, magnesium stearic, and glyceryl dibehenate. Hypromellose was the prime polymer in Brand C. In this formulation, silicon dioxide acts as a glidant, and magnesium as a lubricant. The release pattern suggests that hypromellose provides stability from 0 to 4 h under SGF conditions when in the stomach. This formulation is marketed as a CR-release tablet, yet the release pattern suggests that it aligns with extended-release properties. Over a 24 h period, 100% of the niacin was not released from this formulation, suggesting that complete release would take over 24 or 48 h. Generally, an ER tablet is absorbed within 8–12 h [24], meaning our findings suggest that this formulation may be an intermediate between ER and LA. The mechanism of action of CR allows for the amidation pathway more flexibility in terms of time, which results in a reduction in nicotinuric acid. In some patients, the prolonged release of niacin is associated with hepatic stress [28].

The timed-release formulation is long-acting in the body, similar to a slow or controlled release. Brand D, manufactured by Rugby Laboratories, combined microcrystalline cellulose and hypromellose to provide delayed release characteristics of the capsule, which are compatible with our findings. This formulation included silica, which acts as a glidant, and magnesium, which acts as a lubricant. At a low pH, from 0 to 4 h, the combined polymer interaction of microcrystalline cellulose and hypromellose provided tablet protection, to which a much lower release rate was observed compared to Brand C. This suggests that the more polymers in the formulation, the lower the release rate.

In Brand B, manufactured by Rugby Laboratories, the release pattern was not aligned with its release definition. The 500 mg tablets consisted of gelatin, talc, and starch. Previously, gelatin was used to prepare for the modified release of hard gelatin capsules through a simple and fast monolithic lipid matrix formation method [28]. On the other hand, starch has been used to prepare sustained-release tablets [29]. The release pattern of ER is pH-dependent [30]. In the literature, the highest release rate was at a low pH, while a lower release rate was found at a higher pH. However, in our study, the lowest release rate occurred at a lower pH, and the highest release rate was observed at a higher pH. These data suggest that the formulation of gelatin, talc, and starch keeps the tablet more intact, and thus, a lower release rate is seen under SGF. In the formulation, gelatin is water soluble, meaning an increase in niacin release was observed. In addition, starch is insoluble in water but may be soluble at temperatures of 55–60 degrees. Since the tablet containing starch was in warm media for an extended time in dissolution, this may allow the starch to dissolve at a high pH and cause an increase in niacin release.

There are, indeed, some limitations in our study. In some of the experiments, the precipitation of niacin was documented, which the group is currently investigating further. There are several factors that can affect the release rate of niacin. In this study, the niacin release rate was evaluated under two pH conditions: SGF (pH 1.2) and SIF (pH 6.8). However, other literature has evaluated the niacin release over a range of pH media: 1.2, 4.5, 6.8, and in distilled water [16]. The release of niacin was the lowest at pH 6.8, highest at pH 1.2, and similar at pH 4.5 and distilled water [16]. Salt formation is another factor that may influence the release rate of niacin. The crystalline formation of aripiprazole, an antipsychotic drug, was investigated in SGF and SIF media. The anhydrous form (AA) and the synthesized monohydrate form (MA) displayed varying release patterns. In SGF, both AA and the prepared MA form changed to the HCl salt form. However, in SIF media, the HCl salt form and AA both changed to the monohydrate form [31]. This suggests that salt formation can be related to the low niacin release rate in the IR formulation over 24 h. The SGF and SIF media used in this study did not contain pepsin or pancreatin enzymes. However, these enzymes have been shown to affect the dissolution of various molecules and compounds. For instance, whey casein protein was digested more at pH 3 than at pH 5 [32]. The greater the protein was digested, the higher the absorbance value by detection, and thus, the higher the percentage of drug release. This suggests that if the pepsin or pancreatin enzymes were actually present, a higher niacin release rate may be observed in all four formulations (brands) of niacin, such as Brand A, Brand B, Brand C, and Brand D. The f2 for all the pairs ranged from 2.3 to 20.3 and 9.6 to 35.6 in the SIF and SGF media, respectively. In SGF, the f2 similarity values were 32.4 and 35.6 for Brand B–Brand D and Brand D–Brand C, respectively, whereas in SIF, the f2 similarity values were 20.3 and 19.8 for the same pair of brands of niacin formulations, respectively, indicating that they were not bioequivalent (f2 < 50). The similarity factor results for all commercial brands were slightly consistent, as the results indicated a significant prominent difference in the dissolution profiles of all niacin commercial brands, while in the case of Brand A, the difference in dissolution profile was not the case for all other modified release brands.

## 5. Conclusions

Currently, there are several forms of niacin that exist on the market as OTC products; however, a dissolution study has not yet been conducted on them all. The dissolution study predicts the extent to which the different dosage forms are absorbed, and hence, the bioavailability and efficacy of the drug. Moreover, a release study is an important requirement for all solid oral dosage formulations and is used throughout the development life cycle for product release and stability testing. It is a pivotal analytical test used for detecting physical changes in an active pharmaceutical ingredient and formulated product. This in vitro study aimed to assess the dissolution and release rate of four different brands (A–D) of commercial nonprescription niacin dosage forms. The results showed the brand that followed the expected defined release pattern in vitro. The findings were confirmed by employing similarity factor tests, where all f2 comparison values were below 50 for both the SIF and SGF media.

This work presents the need for future niacin in vivo clinical studies to assess, compare, and analyze the dissolution rates in patients. It explores niacin dependency over a wide range of pH values to understand the release behavior over time and its correlation with drug administration. Health providers should consider the variations in efficacy of the over-the-counter niacin products, and consequently, its impact on clinical therapy. This study documents important variations in the dissolution profiles of current brands; further studies that assess the correlation between niacin’s dissolution data and in vivo efficacy are crucial to optimize the health outcomes of patients.

## Figures and Tables

**Table 1:** Investigated medicinal products (IMPs).

Name	Code	Formulation	Niacin Amount per Product	Lot Number #	Expiration Date	Manufacturer	Excipients
Niacin	Brand-A	IR tablet	250 mg	3245ER2513	06/2020	General Nutrition Corporation (GNC)	Dicalcium phosphate; cellulose; whole brown rice powder (*Oryza sativa*)
ER Niacin	Brand-B	Extended-release tablet	500 mg	184548	03/2021	Rugby Laboratories	Gelatin; sugar; talc; pharmaceutical glaze; starch
SLO Niacin	Brand-C	CR tablet	500 mg	383151	04/2022	Main Pointe Pharmaceuticals, LLC.	Hydromellose; hydrogenated vegetable oil; silicon dioxide; magnesium stearate; glyceryl dibehenate;
TR Niacin	Brand-D	Capsule	1000 mg	191879	04/2021	Rugby Laboratories	Microcrystalline cellulose; Hypromellose; stearic acid; silica; magnesium stearate; pharmaceutical glaze; magnesium silicate

## Data Availability

Supplemental data can be provided upon reasonable request.
